# Effect of Light and Cytokinin Modulators on Adventitious Shooting in *Melia volkensii* Gürke

**DOI:** 10.3390/plants15020322

**Published:** 2026-01-21

**Authors:** Nandini Bhogar Suresh, Lenka Plačková, Karel Doležal, Stefaan P. O. Werbrouck

**Affiliations:** 1 Department of Plants and Crops, Faculty of Bioscience Engineering, Ghent University, 9000 Ghent, Belgium; stefaan.werbrouck@ugent.be; 2 Laboratory of Growth Regulators, Palackỳ University and Institute of Experimental Botany, AS CR, Šlechtitelů 11, 783 71 Olomouc, Czech Republic; lenka.plackova@upol.cz (L.P.); karel.dolezal@upol.cz (K.D.)

**Keywords:** cytokinin homeostasis, cytokinin oxidase/dehydrogenase (CKX), INCYDE, light-emitting diodes (LEDs), phenyladenine (PA), shoot organogenesis, thidiazuron (TDZ)

## Abstract

Adventitious shoot regeneration in woody species is regulated by interactions between plant growth regulators, endogenous hormone metabolism, and environmental cues such as light quality. Here, we investigated the effects of thidiazuron (TDZ) and the cytokinin oxidase/dehydrogenase (CKX) inhibitors INCYDE and phenyladenine (PA), in combination with different light spectra, on morphogenesis in *Melia volkensii* leaf explants. TDZ induced the highest frequencies of callus formation and adventitious shoot regeneration, particularly under white light. INCYDE promoted localized regeneration responses, including activation of dormant meristematic regions in secondary leaf axils, whereas PA showed limited regeneration efficiency. Light quality significantly influenced morphogenesis, with white and blue light favoring organized shoot development, while red and far-red light suppressed shoot regeneration and promoted callus formation. Cytokinin profiling revealed treatment-dependent shifts in endogenous cytokinin composition, most notably in isopentenyladenine (iP)-type cytokinins, which is consistent with altered cytokinin degradation dynamics. Cis-zeatin-type cytokinins were abundant across treatments, likely reflecting regulation associated with in vitro culture conditions. These findings indicate that cytokinin metabolism and light quality jointly influence organogenic competence in *Melia volkensii* Gürke, providing a physiological basis for optimizing regeneration strategies in woody plants. This study provides the first integrated analysis of cytokinin-modulating compounds and light spectra on adventitious shoot regeneration in *Melia volkensii*. The findings establish a physiological basis for improving regeneration protocols in recalcitrant woody species and support future biotechnological applications, including genetic improvement and advanced propagation strategies.

## 1. Introduction

Developing robust and reliable adventitious shoot organogenesis protocols is essential for the application of genetic improvement technologies in woody plants, including genetic transformation, genome editing, mutagenesis, and ploidy manipulation. Adventitious regeneration involves coordinated phases of de-differentiation, re-differentiation, and organ formation, which are processes that are tightly regulated by the balance and interaction of auxins and cytokinins [1,2]. Despite extensive progress in herbaceous model species, many woody plants remain highly recalcitrant to in vitro regeneration, limiting their biotechnological exploitation.

Cytokinins play a central role in regulating cell division, meristem activity, and organogenesis during in vitro development [3]. Endogenous cytokinin levels are controlled by biosynthesis, reversible conjugation, and irreversible degradation mediated by cytokinin oxidase/dehydrogenase (CKX) enzymes [4,5]. Targeting cytokinin metabolism, particularly through modulation of CKX activity, has therefore emerged as an effective strategy to enhance regeneration competence in recalcitrant species.

Several synthetic compounds modulate cytokinin activity through combined mechanisms. Thidiazuron (TDZ), a phenylurea-derived cytokinin-like compound, promotes shoot induction by both inhibiting CKX activity and interfering with cytokinin signaling pathways [6]. INCYDE-Cl (2-chloro-6-(3-methoxyphenyl) aminopurine) is a potent CKX inhibitor that also exhibits weak cytokinin receptor activation [7]. Phenyladenine (PA), the structural core of INCYDE derivatives, similarly combines CKX inhibition with limited cytokinin-like activity [8]. Together, these compounds provide complementary tools to modulate endogenous cytokinin homeostasis and signaling.

In addition to hormonal regulation, light quality acts as a key environmental signal controlling in vitro morphogenesis through well-defined photoreceptor systems. Phytochromes respond primarily to red and far-red light, whereas cryptochromes perceive blue light, together regulating developmental transitions such as meristem activation, organ differentiation, and shoot elongation [9,10]. Blue light has been reported to enhance cytokinin sensitivity and promote shoot organogenesis in several species, while red and far-red light often suppress organized shoot formation and favor callus proliferation [11,12].

*Melia volkensii* Gürke is an ecologically and economically important tree species native to semi-arid regions of East Africa, valued for its high-quality timber, rapid growth, and drought tolerance. Despite its importance, research on this species has largely focused on silvicultural performance, ecological adaptation, and field-based propagation. In contrast, biotechnological approaches, including in vitro culture and regeneration, remain poorly developed. To date, reports addressing adventitious shoot regeneration, hormonal regulation, or cytokinin metabolism in *M. volkensii* are extremely limited or absent. This lack of foundational regeneration protocols represents a major constraint for the application of modern biotechnological tools such as genetic transformation, genome editing, and clonal improvement in this species.

The objective of this study was to investigate how cytokinin-modulating compounds (thidiazuron, INCYDE, and phenyladenine), in combination with defined light spectra, influence morphogenic responses and endogenous cytokinin profiles in *M. volkensii* leaf explants. By integrating morphogenic analysis with cytokinin profiling, this work aims to provide physiological insight into the regulation of organogenic competence and to contribute to the development of strategies for the genetic improvement of this economically and ecologically important tree species.

## 2. Results

### 2.1. Morphogenic Responses of Leaf Explants in Cool Fluorescent White Light

**Figure 1:**
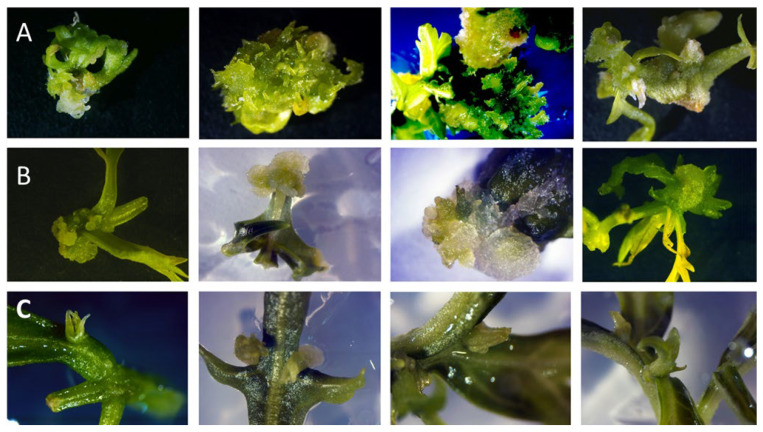
Morphogenic responses of *Melia volkensii* leaf explants to different cytokinin treatments under white light conditions. (**A**) Explants cultured on medium supplemented with thidiazuron (TDZ) showing compact, greenish callus formation and multiple adventitious shoots originating from callus and surrounding leaf tissues. (**B**) Explants cultured on phenyladenine (PA) exhibiting moderate callus formation with limited and sporadic adventitious shoot development. (**C**) Explants cultured on INCYDE displaying localized callus formation and meristematic activity at secondary leaf axils of compound leaves.

Distinct morphogenic responses were observed in the explants throughout the in vitro culture period (Figure 1). Explants cultured on medium supplemented with thidiazuron (TDZ) developed compact, greenish callus tissue with multiple adventitious shoots originating from callus and surrounding leaf tissues (Figure 1A). In contrast, phenyladenine (PA) treatment resulted in moderate callus formation with limited and sporadic shoot development, mainly restricted to leaf margins and axillary regions (Figure 1B). Explants treated with INCYDE displayed localized and compact callus formation, with meristematic activity observed primarily in the secondary leaf axils of compound leaves (Figure 1C).

In contrast, explants treated with PA exhibited moderate callus formation, typically restricted to the leaflet axils and along the leaf margins. Adventitious shoots were initiated but remained few and underdeveloped. The shoots emerged sporadically, often from loosely organized calluses.

Explants exposed to INCYDE showed localized and compact callus formation mainly at axillary regions. Meristematic structures were observed in the secondary leaf axils of compound leaves.

**Figure 2:**
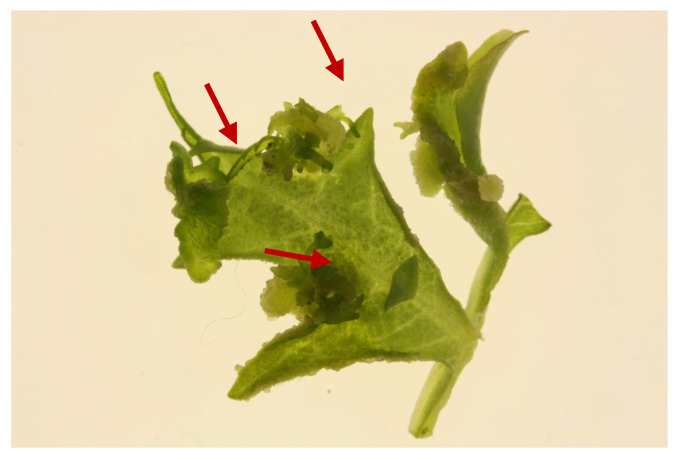
Detailed morphogenic response of *Melia volkensii* leaf explants treated with INCYDE. Localized proliferation of compact callus and adventitious shoot primordia is visible, particularly at leaf margins and secondary leaf axils (Red arrows). Meristematic activity is spatially restricted and does not result in extensive shoot regeneration compared with TDZ-treated explants.

Treatment with INCYDE resulted in the formation of compact, localized callus and limited shoot regeneration compared to TDZ. Reactivation of meristematic structures was observed in the secondary leaf axils of compound leaves (Figure 2).

The effect of the different growth regulator treatments on callus and shoot formation under white light is summarized in Table 1. The hormone-free control produced 0% callus and 0% shoots TDZ showed the highest mean callus induction (23%), followed by INCYDE (20.66%) and PA (14.33%); however, callus formation induced by TDZ and INCYDE was not significantly different, as indicated by the statistical groupings in Table 1. Shoot regeneration followed a similar trend, with TDZ producing 70% shoots, INCYDE 41.66%, and PA 20.33%, while the control produced no shoots. Shoot regeneration in INCYDE-treated explants was spatially restricted to axillary regions, whereas TDZ-induced shoots originated from both callus and surrounding leaf tissues.

### 2.2. Cytokinin Metabolism of Leaf Explants to Different Cytokinin Modulators

Cytokinin profiling was performed on leaf explants without fully developed shoots after six weeks of culture, to minimize the contribution of differentiated shoot tissues to the endogenous hormone measurements. The quantified cytokinin metabolites are presented in Table 2.

Across all samples, cis-zeatin (cZ) and its derivatives (cZR, cZR5′MP, cZROG) represented the largest proportion of detected cytokinins. Although cZ-type cytokinins generally exhibit lower biological activity than trans-zeatin (tZ) or isopentenyladenine (iP) types, they were consistently abundant in both control and treated samples. The high abundance of cZ-type cytokinins was observed irrespective of treatment. The active cytokinin pool consisted mainly of isopentenyladenine (iP) and its derivatives (iPR and iPR5′MP). These compounds were detectable in the control and in all three treatments (INCYDE, PA, and TDZ), with varying levels depending on treatment. Among the measured active CKs, iPR represented the most abundant form.

High levels of meta-topolin riboside (mTR) and meta-topolin (mT) were present in the control, reflecting their use in the medium of the stock plants from which the leaves were taken. After six weeks on media containing INCYDE, PA, or TDZ, there was a strong reduction in both mTR and mT, as well as in their conjugated forms (mT7G, mT9G, oT7G, etc.). Compounds such as BA and oTR-type metabolites, which are derived from mTR pathways, were also present at measurable levels in the control but reduced in the treated samples. The high abundance of meta-topolin-derived cytokinins in control explants primarily reflects residual uptake from the donor plant medium rather than treatment-induced biosynthesis.

A range of O-glucosides and N-glucosides of cZ, DHZ, BAP, mT, and others were detected at low to moderate concentrations depending on treatment. Some metabolites, including tZ7G, tZ9G, iP7G, and iP9G, remained below detection limits in all samples.

Overall, the cytokinin profiles showed clear treatment-dependent differences in the quantities of iP-type cytokinins, mTR-derived cytokinins, and cZ-type compounds after six weeks of culture.

**Figure 3:**
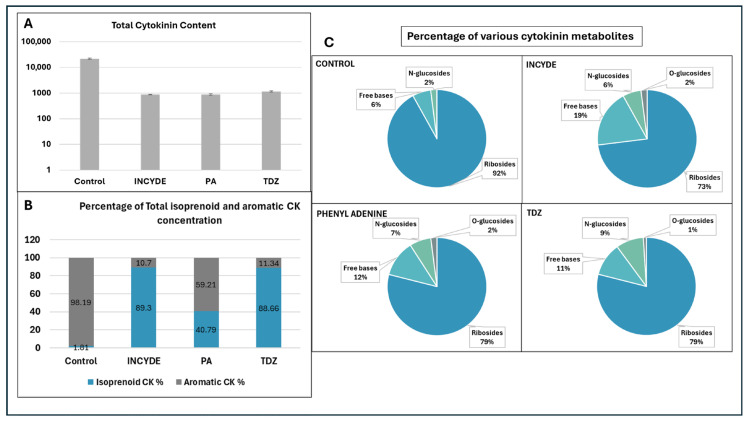
Impact of cytokinin inhibitors and cytokinin analogs on: (**A**): Total cytokinin content, (**B**): cytokinin type distribution, and (**C**): Metabolite composition.

Figure 3 provides an overview of cytokinin distribution patterns in control, INCYDE, PA, and TDZ-treated *Melia volkensii* leaf explants after six weeks of culture. Total cytokinin content was highest in the control explants and markedly reduced in all cytokinin-modulator treatments (Figure 3A). This reduction reflects substantial changes in cytokinin homeostasis following exposure to TDZ, INCYDE, or PA. Analysis of cytokinin classes revealed that isoprenoid cytokinins dominated the cytokinin pool in all treatments, although PA-treated explants showed a relatively higher proportion of aromatic cytokinins compared with the control and other treatments (Figure 3B). The distribution of cytokinin metabolite forms further demonstrated clear treatment-dependent differences, with control explants characterized by a high proportion of ribosides, largely due to the abundance of meta-topolin riboside-derived compounds (Figure 3C).

The elevated levels of meta-topolin-derived cytokinins observed in the control primarily reflect residual uptake from the donor plant medium rather than treatment-induced biosynthesis. Although control explants were maintained on hormone-free medium for six weeks, limited cell division and reduced metabolic activity under these conditions likely minimized dilution of pre-existing topolin pools. In contrast, explants treated with TDZ, INCYDE, or PA underwent active callus formation and cell proliferation, resulting in progressive dilution of residual topolin-derived cytokinins through repeated cell divisions. In addition, cytokinin-modulating treatments are expected to enhance cytokinin turnover via increased metabolic conversion, conjugation, and degradation, further contributing to the depletion of topolin-derived cytokinins in treated explants. Together, these patterns indicate that the observed differences in cytokinin composition among treatments reflect both altered cytokinin metabolism and differences in cellular proliferation dynamics.

**Figure 4:**
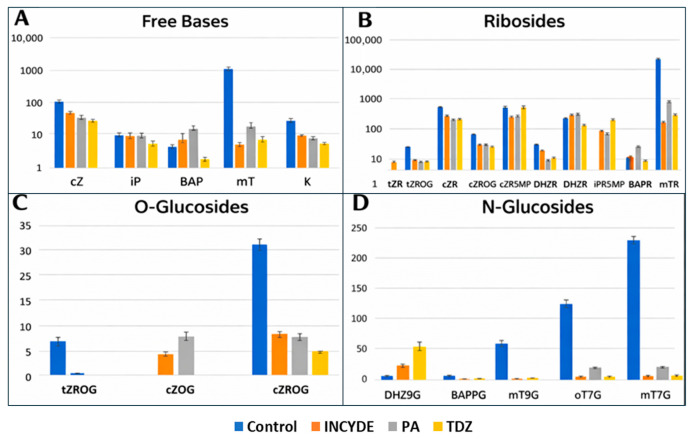
Cytokinin Metabolite Distribution Across Control and Treated Samples (INCYDE, PA, TDZ. (**A**): Free bases (cZ, IP, BAP, mT, oT, K), (**B**): ribosides (tZR, tZROG, cZR, cZROG, cZR5′MP, DHZR, iPR, iPR5′MP, BAPR, mTR), (**C**): O-glucosides (tZROG, cZOG, cZROG), and (**D**): N-glucosides (DHZ9g, BAP9G, mT9G, oT9G, mT7G).

Figure 4 presents a detailed breakdown of individual cytokinin metabolites across control, INCYDE, PA, and TDZ-treated explants after six weeks of culture. The Figure is organized into four panels corresponding to major cytokinin metabolite groups. Figure 4A shows the profiles of free-base cytokinins. In the control explants, several free bases are present, including cZ, iP, BAP, and mT, with mT showing particularly high levels due to its presence in the donor plant medium. In the treated samples, the levels of free bases differ from the control, with visible variation among INCYDE, PA, and TDZ-treated explants. Overall, treated samples show lower concentrations of topolin-derived free bases compared with the control. Figure 4B displays the distribution of riboside forms. In the control, ribosides such as cZR, iPR, and BAPR are present at measurable levels. In the treated samples, the amounts of these ribosides vary across treatments, with reductions observed for topolin-derived ribosides relative to the control. Endogenous ribosides, including cZR and iPR, remain detectable in all treatments, though their relative amounts differ between INCYDE, PA, and TDZ.

Figure 4C presents the O-glucoside cytokinin conjugates. The control samples show prominent levels of topolin O-glucosides such as mT7G and mT9G, reflecting their exogenous origin. In the treated explants, these topolin-derived O-glucosides are reduced compared with the control, while endogenous cZ- and tZ-type O-glucosides remain present at measurable levels across all treatments. Figure 4D shows the N-glucoside conjugates. In the control group, topolin-derived N-glucosides (e.g., mT9G and mT7G) are highly abundant. In all treatments, the concentrations of these topolin-derived compounds are strongly reduced. Other N-glucosides, such as DHZ9G and BAP9G, are present at low amounts and remain detectable across treatments. Together, Figure 3 and Figure 4 reveal treatment-dependent differences in the abundance and distribution of endogenous cytokinin metabolites, as well as the depletion of exogenously derived topolin compounds after six weeks of culture on media containing INCYDE, PA, or TDZ.

### 2.3. Effect of Different Light on Morphogenic Responses of Leaf Explants to Different Cytokinin Modulators

**Figure 5:**
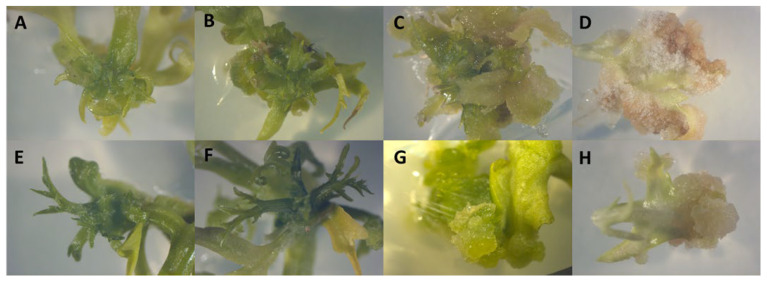
The morphogenic responses of explants treated with two different plant growth regulators, TDZ (Thidiazuron) and INCYDE, under various light spectra. Panels A to D represent explants cultured with TDZ under white (**A**), blue (**B**), red (**C**), and far-red (**D**) light conditions. Notable differences in tissue morphology and regeneration potential can be observed, with varying degrees of callus formation and shoot initiation depending on the light quality. Panels E to H show explants treated with INCYDE under white (**E**), blue (**F**), red (**G**), and far-red (**H**) light. The response to INCYDE also varies with light spectrum, displaying a range of morphogenic outcomes, from organized shoot formation to unorganized or compact calluses.

In this experiment, leaf explants were cultured on media supplemented with either TDZ or INCYDE and exposed to four light spectra—white, blue, red, and far-red—to assess how light quality influenced callus formation and adventitious shoot regeneration (Figure 5). The quantitative responses are summarized in Table 3. Explants treated with TDZ displayed clear differences in morphology across light spectra. Under white light, explants produced 23% callus and 70% adventitious shoots. Under blue light, TDZ-treated explants formed 20.33% callus and 56.33% shoots. Exposure to red light resulted in reduced shoot regeneration (13.33%) and lower callus formation (15.66%). Under far-red light, explants formed 18% callus but 0% shoots.

Explants cultured with INCYDE also showed spectrum-dependent responses. Under white light, explants formed 20.66% callus and 41.66% shoots. Under blue light, callus formation was 17% and shoot regeneration reached 24.33%. Under red light, explants produced 11.33% callus and 3% shoots. As observed with TDZ, far-red light resulted in 0% shoot regeneration, while callus formation remained at 18%.

Under dark (skotomorphogenic) conditions, explants predominantly formed callus tissue and failed to regenerate organized shoots. Notably, TDZ-treated explants exhibited somatic embryo-like structures in 46.33 ± 4.19% of explants, with a subset progressing to early germination stages. No somatic embryogenesis was observed in INCYDE-treated explants under dark conditions.

Across all light conditions, TDZ-treated explants produced higher shoot regeneration and callus formation than INCYDE-treated explants, except under far-red light, for which both regulators yielded 0% shoots. Callus formation did not differ markedly between TDZ and INCYDE under white or far-red light, whereas differences were observed under blue and red light. White light supported the highest shoot induction in both TDZ and INCYDE treatments, while red and far-red light resulted in reduced or absent shoot regeneration. Statistical comparisons were not applied to dark-grown explants due to the qualitative nature of the observed developmental responses.

## 3. Discussion

This study investigated how cytokinin-modulating compounds, in combination with defined light spectra, influence morphogenic responses and endogenous cytokinin profiles in *Melia volkensii* leaf explants. By integrating regeneration outcomes with detailed cytokinin profiling, this work provides new insights into the hormonal and environmental regulation of organogenic competence in a recalcitrant woody species.

### 3.1. Effects of Cytokinin-Modulating Compounds on Morphogenesis

Among the cytokinin-modulating compounds tested, thidiazuron (TDZ) consistently induced the highest frequencies of callus formation and adventitious shoot regeneration. The strong morphogenic response observed under TDZ treatment is in agreement with numerous reports describing TDZ as a highly potent cytokinin-like regulator that promotes shoot organogenesis through combined inhibition of cytokinin oxidase/dehydrogenase activity and interference with cytokinin signaling pathways [13,14]. In *M. volkensii*, TDZ not only enhanced shoot induction but also promoted compact, green callus formation, suggesting sustained cytokinin activity during early developmental stages.

INCYDE, a specific inhibitor of cytokinin degradation, induced a distinct and more localized morphogenic response. The activation of meristematic regions in secondary leaf axils indicates that CKX inhibition can enhance cytokinin availability in tissues that retain latent meristematic competence, as reported previously in other woody perennials [8,15]. However, the overall regeneration efficiency under INCYDE remained lower than that observed with TDZ, suggesting that CKX inhibition alone may be insufficient to overcome broader developmental constraints in *M. volkensii*. Phenyladenine (PA), which exhibits weaker cytokinin activity, resulted in limited regeneration, further emphasizing that both the chemical nature and mode of action of cytokinin modulators critically determine morphogenic outcomes.

### 3.2. Cytokinin Metabolism During Early Organogenic Development

Cytokinin profiling revealed pronounced treatment-dependent differences in endogenous cytokinin composition. Across all treatments, cis-zeatin (cZ)-type cytokinins represented a major fraction of the cytokinin pool. Although cZ-type cytokinins were historically considered less biologically active, accumulating evidence indicates that they play functional roles in stress-related and developmental regulation, particularly under in vitro culture conditions [16,17]. The high abundance of cZ-type cytokinins in control explants may reflect wound- and culture-induced stress responses rather than direct involvement in shoot induction.

In contrast, isopentenyladenine (iP)-type cytokinins showed clear accumulation in explants treated with cytokinin-modulating compounds. Because iP-type cytokinins are primary substrates of CKX enzymes, their increased levels under INCYDE and TDZ treatments are consistent with effective inhibition of cytokinin degradation [5,18]. The elevated levels of iPR observed in treated explants likely contribute to enhanced cytokinin signaling and morphogenic competence, supporting the observed regeneration responses.

The high abundance of meta-topolin-derived cytokinins in control explants can be attributed to residual uptake from the donor plant medium, which contained meta-topolin riboside prior to explant excision. The marked depletion of these compounds in treated explants after six weeks of culture reflects dilution through cell division and enhanced metabolic turnover in actively proliferating tissues rather than treatment-specific biosynthesis. Importantly, despite similar initial topolin backgrounds, TDZ- and INCYDE-treated explants displayed markedly different morphogenic outcomes, indicating that the applied cytokinin modulators, rather than residual topolins, were the primary drivers of regeneration.

### 3.3. Influence of Light Quality on Regeneration Responses

Light quality exerted a strong modulatory effect on morphogenic responses in *M. volkensii*. White and blue light supported the highest frequencies of organized shoot regeneration, particularly in combination with TDZ. Blue light has been reported to enhance cytokinin sensitivity and promote photomorphogenic development through cryptochrome-mediated signaling pathways [11,12], which is consistent with the greener tissues and improved shoot formation observed in this study.

In contrast, red and far-red light markedly suppressed shoot regeneration while promoting callus formation. Such responses are commonly attributed to phytochrome-mediated regulation of hormonal balance and developmental pathways, where a Pr-dominant state disfavors shoot organogenesis [19,20]. The complete inhibition of shoot formation under far-red light highlights the critical role of light perception in maintaining organogenic competence in woody explants.

An additional observation of interest was the formation of somatic embryo-like structures in TDZ-treated explants under dark conditions. Similar developmental redirection under skotomorphogenic conditions has been reported in other woody species and may reflect suppression of shoot identity pathways combined with strong cytokinin stimulation [21]. Although not explored further here, this response suggests developmental plasticity in *M. volkensii* and warrants future investigation.

### 3.4. Novelty, Practical Significance, and Comparison with Other Woody Species

The present study represents the first integrated analysis of cytokinin-modulating compounds and light spectra on adventitious shoot regeneration and endogenous cytokinin profiles in *Melia volkensii*. While cytokinin–light interactions have been extensively studied in herbaceous and model species, comparable mechanistic studies in recalcitrant woody plants remain limited [11,20]. Most previous reports in woody species have focused on morphogenic outcomes without linking regeneration responses to underlying cytokinin metabolism [2,5].

From a practical perspective, the identification of TDZ as a highly effective inducer of adventitious shoot regeneration under specific light conditions provides a foundation for developing reliable regeneration protocols in *M. volkensii*. Such protocols are essential prerequisites for advanced biotechnological applications, including genetic transformation, genome editing, and clonal improvement of elite genotypes [21,22]. The localized meristematic activation observed with INCYDE further suggests that targeted modulation of cytokinin degradation may be exploited to fine-tune regeneration responses while potentially minimizing abnormal morphogenesis.

When compared with other woody species such as Populus, Eucalyptus, and Pinus, the dependence of regeneration efficiency on cytokinin type and light quality observed in *M. volkensii* is broadly consistent with previous findings [20,21,22]. However, the pronounced dominance of cis-zeatin-type cytokinins and the strong interaction between cytokinin modulators and light spectra highlight species-specific regulatory features that have not been previously reported. These results emphasize the need for species-tailored regeneration strategies rather than reliance on generalized protocols across woody taxa.

The selection of *Melia volkensii* for this study was driven by its relevance to climate-resilient forestry and sustainable timber production. As a drought-tolerant species adapted to semi-arid environments, *M. volkensii* represents an important model for improving tree productivity under increasing climatic stress. The development of reliable regeneration protocols in this recalcitrant species not only facilitates its genetic improvement but also provides insights applicable to other drought-adapted woody taxa with similar propagation constraints.

## 4. Materials and Methods

### 4.1. Plant Materials, Culture Media and Growth Conditions

*M. volkensii* in vitro stock plants were maintained on basal medium consisting of MS macro and micro salts and vitamins [23], with 20 g/L sucrose, 7 g/L Plant Agar (Duchefa, Haarlem, The Netherlands). To this medium, 10 µM meta-topolin riboside (mTR) (Olchemim, Olomouc, Czech Republic) was added as meta-topolin riboside is considered thermostable under standard autoclaving conditions used for plant tissue culture. The pH of the medium was adjusted to 5.4 prior to autoclaving, as preliminary optimization for *Melia volkensii* stock cultures indicated improved explant growth and reduced tissue browning at pH 5.4 compared with the commonly used pH 5.8. This stock was incubated in a growth chamber with 22 °C, with cool fluorescent white light (Philips, Eindhoven, The Netherlands) at an intensity of 40 μmol m^−2^ s^−1^ and a 16-h photo period. Leaves from this in vitro culture were used for all the further experiments.

### 4.2. Adventitious Shoots Induction

Five leaf explants were inoculated in Petri dishes containing 10 mL basal medium with 10 µM TDZ, 10 µM INCYDE or 10 µM PA. The control treatment did not contain plant growth regulators. INCYDE was prepared as described by [15]. Plates were incubated under the same conditions as the stock plants. We recorded the number of explants with callus after 3 weeks and the number with shoots after 6 weeks.

### 4.3. Effect of Light Quality

Leaf explants were inoculated in Petri dishes containing 10 mL of basal medium supplemented with either 10 µM TDZ or 10 µM INCYDE. The effects of cool fluorescent white light were compared with blue light (454 nm), red light (660 nm), far-red light (745 nm), and darkness under a 16 h photo-period. Except for far-red light, all light treatments were applied at an intensity of 40 μmol m^−2^ s^−1^, measured using a JAZ light meter (Ocean Optics, Orlando, FL, USA). The Petri dishes were placed in boxes with white walls and a white door in which Philips GreenPower LED Research Modules (Philips, Eindhoven, The Netherlands) were mounted. For dark treatments, Petri dishes were wrapped in two layers of aluminum foil immediately after inoculation and incubated in a light-proof growth chamber. Plates were unwrapped only briefly during subculturing and scoring, which were conducted under dim green safe light to maintain dark conditions while allowing minimal handling and gas exchange. After three weeks of culture, the number of explants forming callus was recorded, and after six weeks, the number of explants forming adventitious shoots and somatic embryo-like structures was quantified.

### 4.4. Extraction, Purification and Quantification of Endogenous Phytohormones

Cytokinin profiling was performed on leaf explants without fully developed shoots after six weeks of culture to minimize the contribution of differentiated shoot tissues to endogenous hormone measurements. Leaf explants (50) proliferating for 6 weeks on medium with different cytokinins were frozen at −80% for 15 min and lyophilized. The plant material was homogenized and weighed in technical triplicates, containing approximately 0.5 mg DW. Samples were extracted with 0.5 mL of Bieleski buffer and to each sample were added a mix of isotope-labelled CK internal standards (0.25 pmol per sample of B, R, 7G, 9G and 0.5 pmol of OG, NT). Purification was performed using StageTips method described in [24]. Samples were analysed by ultraperformance liquid chromatography (Acquity UPLC^®^I-class system; Waters, Milford, MA, USA) coupled to a triple quadrupole mass spectrometer equipped with an electrospray interface (Xevo TQ-S, Waters, Manchester, UK) by a method described in [24]. Quantification was obtained by multiple reaction monitoring of [M + H] and the appropriate product ion. Optimal conditions, dwell time, cone voltage and collision energy in the collision cell were optimized for each CK metabolite [24,25]. For each treatment, explants were derived from multiple independently propagated in vitro donor plants to ensure biological replication, while hormone extraction and quantification were performed in technical triplicates.

### 4.5. Statistical Analysis

The experiment was carried out with 25 explants and repeated three times. Data was analyzed using SPSS 25.0 (SPSS Inc., Chicago, IL, USA). The data were analyzed by analysis of variance (ANOVA) followed by Duncan’s new multiple range (DMR) test to separate the mean differences [26]. For cytokinin analysis, samples consisted of pooled explants derived from independent in vitro donor plants, whereas morphogenic responses were analyzed at the individual explant level. Dark-grown explants were excluded from statistical comparison due to qualitative differences in developmental responses.

## 5. Conclusions

This research underscores the complex and highly regulated nature of plant in vitro development, where intricate interactions between exogenous PGRs, endogenous hormone profiles, and environmental cues such as light quality dictate morphogenic outcomes. While TDZ remains a highly effective broad-spectrum cytokinin for regeneration, INCYDE offers a distinct mode of action by modulating endogenous cytokinin metabolism, which may facilitate localized meristem activation under favorable conditions. PA, while affecting the cytokinin profile, proves less efficient for robust regeneration. The observed shifts in cytokinin profiles, particularly the accumulation of iP-type cytokinins, are consistent with altered cytokinin degradation dynamics following treatment. The profound impact of light quality, particularly the inhibitory effects of red and far-red light on organized shoot development, emphasizes the critical involvement of photoreceptors in regulating in vitro developmental pathways.

Future research could build on these findings with transcriptomic and proteomic approaches to see how different light spectra modulate the expression of cytokinin-related genes (e.g., IPT, CKX) and signaling components. Such studies would clarify how light fine-tunes the plant’s hormonal response to the applied growth regulators. Understanding these molecular interactions will pave the way for designing more efficient and tailored tissue culture protocols, enabling precise control over plant growth and development for various biotechnological applications.

## Figures and Tables

**Table 1:** The effect of hormone free, TDZ, INCYDE, and PA on % of leaves with callus and shoot formation under fluorescent light conditions. Data are presented as mean percentages ± standard error (SE). Means followed by the same letter are not significantly different (Duncan, *p* < 0.05). Different superscript letters (a, b, c, d) indicate significant differences between treatments within each row, based on a one-way ANOVA followed by Duncan’s post hoc test (*p* < 0.05).

	Control	TDZ	INCYDE	PA
Callus	0.00 ^a^	23.00 ± 0.57 ^b^	20.66 ± 0.88 ^bc^	14.33 ± 0.88 ^c^
Shoot	0.00 ^a^	70.00 ± 1.53 ^b^	41.66 ± 1.76 ^c^	20.33 ± 2.60 ^d^

**Table 2:** Quantification of various cytokinins and their metabolites in *M. volkensii* leaf explants incubated for 6 weeks on medium with INCYDE, PA, TDZ, or PGR-free medium. Values are expressed as mean ± standard deviation. LOD indicates levels below the limit of detection. All abbreviations used in this table are defined in the Abbreviations section at the end of the manuscript.

	Control	INCYDE-Cl	PA	TDZ
tZ	<LOD	<LOD	<LOD	<LOD
tZOG	<LOD	<LOD	<LOD	<LOD
tZR	1.35 ± 0.02	1.61 ± 0.04	0.76 ± 0.02	0.37 ± 0.03
tZROG	7.41 ± 0.50	<LOD	<LOD	<LOD
tZ7G	<LOD	<LOD	<LOD	<LOD
tZ9G	<LOD	<LOD	<LOD	<LOD
tZR5′MP	<LOD	<LOD	<LOD	<LOD
cZ	150.47 ± 0.13	68.97 ± 8.58	30.27 ± 4.19	49.02 ± 0.83
cZOG	<LOD	3.07 ± 0.16	8.22 ± 0.52	<LOD
cZR	214.65 ± 6.41	113.32 ± 1.48	76.40 ± 0.14	77.68 ± 7.08
cZROG	29.46 ± 1.54	8.08 ± 0.18	7.40 ± 0.26	5.59 ± 0.09
cZ7G	<LOD	<LOD	<LOD	<LOD
cZ9G	<LOD	<LOD	<LOD	<LOD
cZR5′MP	268.84 ± 44.87	105.41 ± 11.25	84.10 ± 26.47	245.01 ± 36.74
DHZ	<LOD	<LOD	<LOD	<LOD
DHZOG	<LOD	<LOD	<LOD	<LOD
DHZR	6.35 ± 0.12	4.44 ± 0.20	2.21 ± 0.21	2.00 ± 0.24
DHZROG	<LOD	<LOD	<LOD	<LOD
DHZ7G	<LOD	<LOD	<LOD	<LOD
DHZ9G	5.58 ± 0.21	24.17 ± 1.37	15.48 ± 0.73	55.26 ± 6.44
DHZR5′MP	<LOD	<LOD	<LOD	<LOD
iP	14.86 ± 0.76	12.94 ± 1.67	12.42 ± 1.78	7.98 ± 1.07
iPR	60.48 ± 2.63	107.00 ± 2.15	111.94 ± 7.77	63.10 ± 6.15
iP7G	<LOD	<LOD	<LOD	<LOD
iP9G	<LOD	<LOD	<LOD	<LOD
iPR5′MP	<LOD	23.55 ± 1.12	16.64 ± 6.32	59.80 ± 5.75
BAP	6.06 ± 1.38	12.56 ± 2.11	23.33 ± 3.95	2.03 ± 0.45
BAPR	1.81 ± 0.14	2.54 ± 0.13	6.04 ± 0.25	1.69 ± 0.12
BAP7G	<LOD	<LOD	<LOD	<LOD
BAP9G	<LOD	<LOD	1.28 ± 0.10	<LOD
BAPR5′MP	<LOD	<LOD	<LOD	<LOD
mT	1087.00 ± 77.96	5.74 ± 0.44	32.59 ± 3.89	10.77 ± 1.23
mTR	19,912.27 ± 1025.00	59.38 ± 4.17	414.03 ± 6.45	104.26 ± 4.14
mT7G	223.49 ± 3.00	6.72 ± 0.18	17.88 ± 0.36	5.65 ± 0.14
mT9G	58.84 ± 1.01	<LOD	<LOD	<LOD
oT	<LOD	<LOD	<LOD	<LOD
oTR	<LOD	<LOD	<LOD	<LOD
oT7G	118.57 ± 6.28	6.89 ± 0.26	25.71 ± 1.17	7.44 ± 0.18
oT9G	<LOD	<LOD	<LOD	<LOD
pT	<LOD	<LOD	<LOD	<LOD
pTR	<LOD	<LOD	<LOD	<LOD
pT7G	<LOD	<LOD	<LOD	<LOD
pT9G	<LOD	<LOD	<LOD	<LOD
K	44.16 ± 5.50	11.32 ± 0.21	9.82 ± 1.39	6.14 ± 0.02
KR	<LOD	<LOD	<LOD	<LOD
K9G	<LOD	<LOD	<LOD	<LOD

**Table 3:** The effects of different light spectra (Dark, White, Blue, Red, and Far-Red) on callus induction and adventitious shoot regeneration in the presence of two plant growth regulators: TDZ (Thidiazuron) and INCYDE. Data are presented as mean values ± standard error. Superscript letters indicate statistical differences between treatments (*p* ≤ 0.05) based on appropriate post hoc tests. Treatments sharing at least one common letter are not significantly different from each other. Statistical comparison was not applied to dark-grown explants.

		TDZ	INCYDE
White light	Callus	23 ± 0.82 ^a^	20.66 ± 1.25 ^a,b^
	Adv shoots	70 ± 2.16 ^a^	41.66 ± 2.49 ^c^
Blue Light	Callus	20.33 ± 1.25 ^a,b^	17 ± 0.82 ^c^
	Adv shoots	56.33 ± 4.5 ^b^	24.33 ± 2.87 ^d^
Red Light	Callus	15.66 ± 1.7 ^d^	11.33 ± 1.70 ^e^
	Adv shoots	13.33 ± 1.25 ^e^	3 ± 0.82 ^f^
Far Red Light	Callus	18 ± 1.63 ^b,c^	18 ± 0.82 ^b,c^
	Adv shoots	0.00 ^g^	0.00 ^g^
Dark	Callus	22.33 ± 1.25	23 ± 0.82
	Somatic embryo	46.33 ± 4.19	0.00
	Germination of SE	13.33 ± 1.25	0.00

## Data Availability

Data will be made available on request.

## Abbreviations

The following abbreviations are used in this manuscript:

PGRs	Plant Growth Regulators
CKX	Cytokinin Oxidases/Dehydrogenase
TDZ	Thidiazuron (synthetic cytokinin-like phenylurea used in tissue culture)
MS	Murashige and Skoog medium
mTR	meta-Topolin Riboside (aromatic cytokinin)
mT	meta-Topolin (natural cytokinin free base)
PA	Phenyladenine (synthetic aromatic cytokinin analog, core molecule of INCYDE derivatives)
INCYDE	2-chloro-6-(3-methoxyphenyl)aminopurine (CKX inhibitor)
cZ	cis-Zeatin (natural cytokinin free base isomer)
tZ	trans-Zeatin (natural cytokinin free base)
DHZ	Dihydrozeatin (natural cytokinin free base)
iP	Isopentenyladenine (natural cytokinin free base)
BAP	Benzylaminopurine (synthetic cytokinin, also called 6-benzylaminopurine)
oT	ortho-Topolin (natural cytokinin free base)
pT	para-Topolin (natural cytokinin free base)
K	Kinetin (natural cytokinin free base)
LOD	Limit of Detection
ANOVA	Analysis of Variance
SE	Standard Error
PAR	Photosynthetically Active Radiation
LED	Light Emitting Diodes
tZOG	trans-Zeatin O-glucoside (O-glucoside conjugate of trans-zeatin)
tZR	trans-Zeatin Riboside (transport form of cytokinin)
tZROG	trans-Zeatin Riboside O-glucoside
tZ7G	trans-Zeatin 7-glucoside (glucoside conjugate)
tZ9G	trans-Zeatin 9-glucoside (glucoside conjugate)
tZR5′MP	trans-Zeatin Riboside 5′-monophosphate (cytokinin nucleotide)
cZOG	cis-Zeatin O-glucoside
cZR	cis-Zeatin Riboside
cZROG	cis-Zeatin Riboside O-glucoside
cZ7G	cis-Zeatin 7-glucoside
cZ9G	cis-Zeatin 9-glucoside
cZR5′MP	cis-Zeatin Riboside 5′-monophosphate
DHZOG	Dihydrozeatin O-glucoside
DHZR	Dihydrozeatin Riboside
DHZROG	Dihydrozeatin Riboside O-glucoside
DHZ7G	Dihydrozeatin 7-glucoside
DHZ9G	Dihydrozeatin 9-glucoside
DHZR5′MP	Dihydrozeatin Riboside 5′-monophosphate
iPR	Isopentenyladenine Riboside
iP7G	Isopentenyladenine 7-glucoside
iP9G	Isopentenyladenine 9-glucoside
iPR5′MP	Isopentenyladenine Riboside 5′-monophosphate
BAPR	Benzylaminopurine Riboside
BAP7G	Benzylaminopurine 7-glucoside
BAP9G	Benzylaminopurine 9-glucoside
BAPR5′MP	Benzylaminopurine Riboside 5′-monophosphate
mT7G	meta-Topolin 7-glucoside
mT9G	meta-Topolin 9-glucoside
oTR	ortho-Topolin Riboside
oT7G	ortho-Topolin 7-glucoside
oT9G	ortho-Topolin 9-glucoside
pTR	para-Topolin Riboside
pT7G	para-Topolin 7-glucoside
pT9G	para-Topolin 9-glucoside
KR	Kinetin Riboside
K9G	Kinetin 9-glucoside
Control	Untreated control group (baseline)
CK	Cytokinins
IPT	Isopentenyl Transferase (cytokinin biosynthesis gene)
PPFD	Photosynthetic Photon Flux Density
FW	Fresh Weight
